# Antioxidant activation, cell wall reinforcement, and reactive oxygen species regulation promote resistance to waterlogging stress in hot pepper (*Capsicum annuum* L.)

**DOI:** 10.1186/s12870-022-03807-2

**Published:** 2022-09-01

**Authors:** Xuefeng Gong, Yi Xu, Hong Li, Xin Chen, Zhanfeng Song

**Affiliations:** 1 Horticulture Institute, Sichuan Academy of Agricultural Sciences, Chengdu, 610066 China; 2 Vegetable Germplasm Innovation and Variety Improvement Key Laboratory of Sichuan Province, Chengdu, 610066 China; 3 Key Laboratory of Horticultural Crops Biology and Germplasm Enhancement in Southwest Regions, Ministry of Agriculture in Rural Affairs of the P.R. China, Chengdu, 610066 China

**Keywords:** Hot pepper, *Capsicum annuum* L., Waterlogging, RNA-seq, Antioxidant enzymes

## Abstract

**Background:**

Hot pepper (*Capsicum annuum* L.) is one of the world’s oldest domesticated crops. It has poor waterlogging tolerance, and flooding frequently results in plant death and yield reduction. Therefore, understanding the molecular mechanisms associated with pepper waterlogging tolerance is essential to grow new varieties with stronger tolerance.

**Results:**

In this study, we discovered that after 5 days of flooding, the growth rate of waterlogging-tolerant pepper cultivars did not reduce to a large extent. Physiological data revealed that chlorophyll concentration was not significantly affected by flooding; however, stomatal conductance was altered considerably 0–5 days after flooding, and the net photosynthesis rate changed substantially 5–10 days after flooding. In addition, the root activity of waterlogging-tolerant varieties was substantially higher after flooding for 10 days than that of the control. This implies that the effect of flooding is associated with changes in the root environment, which ultimately affects photosynthesis. We evaluated changes in gene expression levels between two pepper types at the same time point and the same pepper variety at different time points after flooding stress treatment and performed a screening for multiple potential genes. These differentially expressed genes (DEGs) were further analyzed for functional enrichment, and the results revealed that antioxidase genes, cell wall synthesis pathway genes, and calcium ion regulation pathway genes might be associated with waterlogging tolerance. Other genes identified in peppers with waterlogging tolerance included those associated with lignin synthesis regulation, reactive oxygen species (ROS) regulation pathways, and others associated with stress resistance. Considerable changes in the expression levels of these genes were recorded 5 days after waterlogging, which was consistent with a considerable increase in oxidase content that was also noted on the fifth day after flooding. The quantitative reverse transcriptase polymerase chain reaction (qRT-PCR) findings revealed that among the 20 selected DEGs, including genes such as *mitogen-activated protein kinase 3 (MPK3)* and *calcium-binding protein 4 (CML4),* approximately 80% of the gene expression patterns were consistent with our RNA-seq dataset.

**Conclusions:**

The findings of this study suggest that ROS modulation, increased antioxidase activity, lignin formation, and the expression of stress resistance genes help peppers with waterlogging tolerance resist flooding stress in the early stages. These findings provide a basis for further investigation of the molecular mechanisms responsible for waterlogging tolerance in pepper and may be a critical reference for the breeding of hot pepper.

**Supplementary Information:**

The online version contains supplementary material available at 10.1186/s12870-022-03807-2.

## Background

Plant growth and development are influenced by the availability of water. Plant metabolism and morphogenesis require sufficient water, but excess of water can disrupt the water balance, thereby influencing plant metabolism, nutrient absorption, and oxygen exchange, and eventually compromising plant growth, development, yield, and quality [1, 2]. Waterlogging damage to plants is caused indirectly because immersion causes the loss of mineral elements and valuable intermediate products in the root system. More so, waterlogging results in the buildup of toxic substances, such as acetaldehyde and ethanol, generated by anaerobic respiration. Furthermore, excess of water restricts plant root growth [3–5]. In addition, soil with excess water causes oxygen deficiency and carbon dioxide and ethylene surplus in plants; this can result in hypoxia, which triggers a series of plant stress reactions. Hypoxia causes a breakdown in the equilibrium of intracellular free radical formation, scavenging, and accumulation of free radicals, which damages membrane structure and function while also increasing membrane permeability [3, 4].

Hot pepper (*Capsicum* spp.) is an annual or perennial herb belonging to the Solanaceae family. Pepper is a plant that is native to Mexico, South America, and the West Indies, but it is now grown throughout the world, including Asia, Africa, and Europe. Currently, it is the second most widely cultivated nightshade vegetable crop in the world after tomato [6, 7]. More so, it is the principal spice used in northwest and southwest China, with one of the largest planting areas and highest economic worth of any vegetable crop in China. Peppers are extremely nutritious, containing the highest vitamin C content among all vegetables [8]. Furthermore, peppers are a source of plant-derived compounds with non-food applications, including green chemicals and antimicrobial materials, both of which have high growth potential [9–11].

Peppers are neither drought- nor water-tolerant [2]. Waterlogging stress can severely limit the normal growth of pepper and thus affects the development of commercial pepper production. Pepper is a shallow-rooted plant that is tolerant to neither drought nor flood, thereby making it ideal for growing in dry climates [7]. Pepper is grown in open fields in most parts of China. Below the middle and lower reaches of the Yangtze River, damage caused by waterlogging is noted relatively often, which considerably affects the yield and quality of the grown peppers. Excess soil moisture has various effects on pepper growth and photosynthesis, and flooding spanning a period of several hours can result in plant mortality [2, 3]. Many types of researches have been conducted on the physiological changes in crops, including wheat [12], maize [13, 14], rapeseed [15], and peanut [16, 17], in response to waterlogging. In tomatoes, treatment of waterlogged seedlings with benzyladenine relieved most symptoms of flooding injury [18]. Ethylene synthesis can be exacerbated by waterlogging Previous research has revealed that 1-aminocyclopropane-1-carboxylic acid, the immediate precursor of ethylene, is produced in the anaerobic root and transported to the shoot and then readily transformed into ethylene [19]. Waterlogging also increased alcohol dehydrogenase (ADH) activity and decreased abscisic acid (ABA) levels while inducing ABA receptors and ABA-dependent transcription factor transcripts [20, 21]. Micro RNAs may also be involved in stress response and affect root development and limit root length [22]. However, research on the molecular mechanisms involved in the ability of pepper to resist waterlogging has been scanty. In this study, RNA-seq was performed to identify these molecular mechanisms in hot pepper. Two pepper varieties with different waterlogging resistances were subjected to three different waterlogging treatment periods to produce mRNA expression profiles. DEGs were then compared between the varieties and treatments and this data set was combined with additional functional, physiological, and biochemical analyses. This facilitated the identification of genes that may be linked to the molecular modulation of waterlogging resistance in pepper. These results provide a novel insight into the molecular basis of waterlogging tolerance in pepper, which is necessary for the development of stress-tolerant germplasm in the future.

## Results

### Variation in the waterlogging resistance of the two varieties

**Fig. 1:**
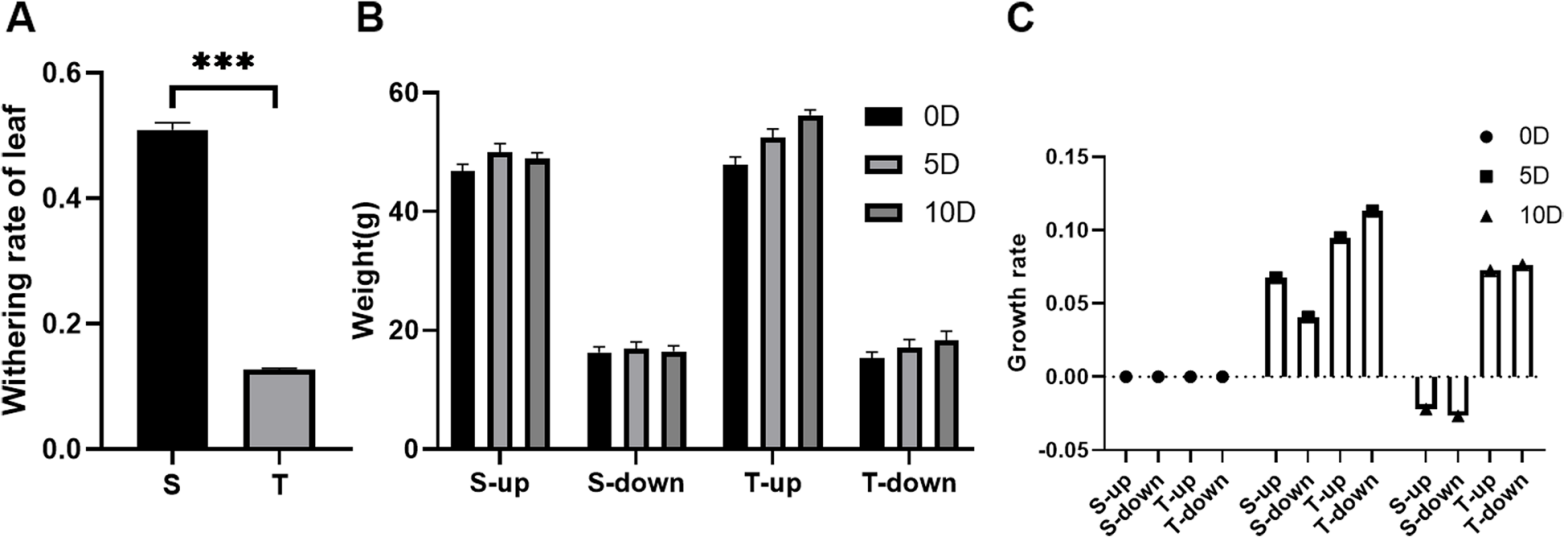
The phenotypic differences of two pepper varieties to flooding. **A** Plant leaf withering rate at 10 days after waterlogging treatment **B** Weight change of the plant **C** Growth rate of different parts of the plant. S, *Chuan Teng No.6; T, Chuan Teng No.10; 0D-10D, 0-10Day. Up,* Aboveground part of the plant; down, underground part of the plant. Error bars indicate the SD (*n* ≥ 3) of three biological replicates. Asterisks indicate statistically significant differences as determined by Student’s *t*-test (*P < 0.05, **P < 0.01, ***P < 0.001)

This study compared the waterlogging resistance of the waterlogging-sensitive “Chuan Teng No.6” (S) hot pepper variety and the waterlogging-tolerant “Chuan Teng No.10” (T) variety. The phenotypic differences between the two pepper varieties were initially assessed to screen for genes associated with flooding resistance after waterlogging. The results revealed that the leaf wilting rate of S was nearly 50% after a waterlogging treatment of 10 d, whereas the leaf wilting rate of T was approximately 10%; therefore, the waterlogging tolerance properties of the two varieties were found to be considerably different (Fig. 1A). Statistical analysis of plant weight following waterlogging showed that the weight of S gradually increased at 5 days after treatment (dat) but decreased at10 dat. T, in contrast, was less affected by waterlogging, and its weight gradually increased throughout the treatment (Fig. 1B). Furthermore, when the growth rate of the entire plant was calculated, S was found to have a lower rate than T at 5 dat, although the weight of S decreased as a result of wilting at 10 dat. Conversely, the growth rate of T was lower at 10 dat than at 5 dat, but its weight still showed a gradual increase (Fig. 1C). Conclusively, these findings suggested that the two pepper varieties had considerable differences in waterlogging resistance and had different molecular regulatory mechanisms.

### RNA sequencing of the two varieties

RNA samples obtained from the roots of both S and T varieties under control and waterlogging treatment conditions were obtained at 0, 5, and 10 dat. These samples were sequenced using Illumina sequencing. Approximately 49 million raw reads and 48.9 million clean reads (yielding a clean: raw ratio of nearly 99.8%) were obtained from each sample (Table S1). The mean GC content was 42.15%. Following filtering, the Q20 and Q30 were equal to or greater than 97.65% and 93.22%, respectively (Table S2). Clean reads of all samples were then mapped to a ribosomal RNA (rRNA) database. Following the removal of rRNA reads, the remaining ~ 98% unmapped reads for the major samples were further mapped to the pepper reference genome 'Zunla-1' (version 2.0) (Table S3). The findings revealed that an average of 92.9% of reads were mapped to the reference genome (Table S4). Of these, approximately 72% were mapped to exons, 5% to introns, and 23% to intergenic regions (Table S5). Following the mapping and reconstruction of transcripts, 40,540 known genes and 5,204 novel genes were obtained and used in computing the fragment per kilobase of transcript per million mapped (FPKM) read values of each sample.

### Differentially expressed genes (DEGs) between waterlogging-sensitive and -tolerant varieties

**Fig. 2:**
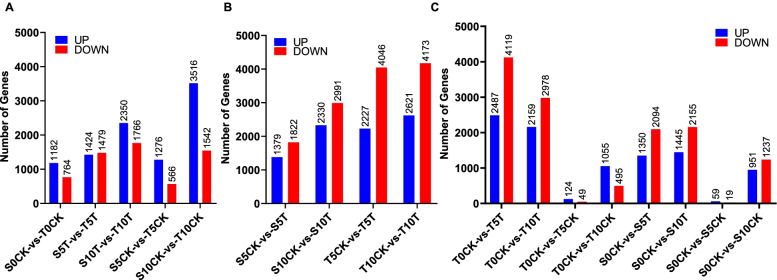
Number of DEGs among the time points of waterlogging treatment in S and T. **A** The DEGs between S and T at three time points both treatment and control. **B** The DEGs between CK and treatment after waterlogging were 5 days and 10 days. **C** The DEGs between three time points

A core set of DEGs at the three time points of the waterlogging stress treatment in the two pepper varieties was identified using edgeR software. Results identified 1,946 DEGs (including 1,182 upregulated genes and 764 downregulated genes) between the two pepper varieties (S0CK and T0CK) before waterlogging, implying that the genetic backgrounds of the two pepper varieties vary (Fig. 2A). After 5 days, the number of DEGs between S5CK and T5CK was similar (1,842 DEGs, including 1,276 upregulated genes and 566 downregulated genes) (Fig. 2A). Nevertheless, major differences in the number of DEGs between S10CK and T10CK at 10 dat (5,058 DEGs, including 3,516 upregulated genes and 1,542 downregulated genes) were noted (Fig. 2A). Furthermore, the number of DEGs gradually increased at 5 dat and 10 dat after the waterlogging treatment ended. Approximately 2,903 DEGs (including 1,424 upregulated genes and 1,479 downregulated genes) were found between S5T and T5T (at 5 dat) (Fig. 2A). Furthermore, 4,116 DEGs (including 2,350 upregulated genes and 1,766 downregulated genes) were found between S10T and T10T (at 10 dat) (Fig. 2A). Additional gene expression comparisons before and after the waterlogging treatment showed 3,201 DEGs for peppers that were sensitive to waterlogging (including 1,379 upregulated genes and 1,822 downregulated genes) prior to the treatment. In addition, 5,321 DEGs (including 2,330 upregulated genes and 2,991 downregulated genes) were found at 5 dat and 10 dat, respectively. For resistant pepper varieties, the numbers of DEGs between the control and treatment identified at 5 dat and 10 dat were 6,273 (2,227 upregulated and 4,046 downregulated) and 6,794 (2,621 upregulated and 4,173 downregulated). Overall, these findings showed a gradual increase in the number of DEGs after waterlogging treatment in both peppers that were sensitive and tolerant to waterlogging (Fig. 2B).

Next, we compared gene expression levels over time. For the waterlogging-tolerant pepper control group, 173 DEGs were found at 5 dat (124 upregulated and 49 downregulated) and 1,550 DEGs at the 10 dat (1,055 upregulated and 495 downregulated) (Fig. 2C). As for the waterlogging-sensitive pepper control group, 78 DEGs were found at 5 dat (59 upregulated and 19 downregulated) and 2,188 DEGs at 10 dat (951 upregulated and 1,237 downregulated) (Fig. 2C). However, more DEGs were noted in the treatment group. At 5 dat (T0CKvsT5T), the number of DEGs observed in waterlogging-tolerant peppers was 6,606 (2,487 upregulated and 4,119 downregulated) and at 10 dat (T0CKvsT10T), 5,137 DEGs were identified (2,159 upregulated and 2,978 downregulated) (Fig. 2C). In waterlogging-sensitive peppers, the number of DEGs noted was 3,444 at 5 dat (S0CKvsS5T; including 1,350 upregulated and 2,094 downregulated genes) and 3,600 DEGs at 10 dat (S0CKvsS10T; including 1,445 upregulated and 2,155 downregulated genes) (Fig. 2C). Overall, these findings showed that the effect of flooding treatment on gene regulation might be greater than the change in growth and development.

### Identification of DEGs involved in the response of pepper plants to waterlogging stress

**Fig. 3:**
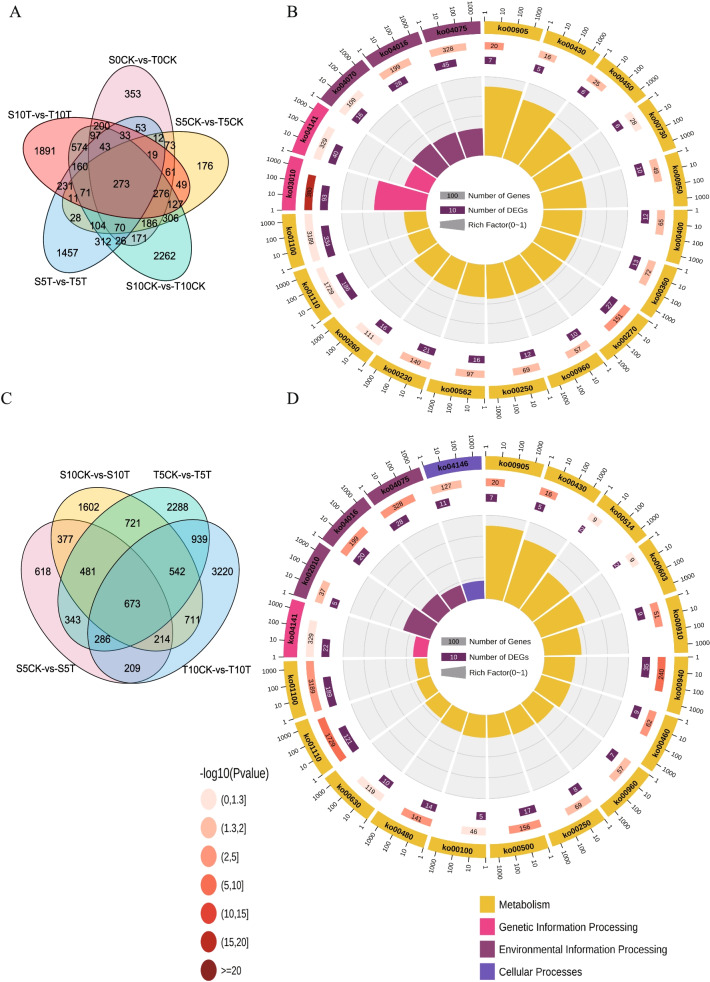
Venn diagram of the comparison groups. **A** Lists of statistically significant DEGs between S and T at three time points both treatment and control were used to create a Venn diagram. **B** Kyoto Encyclopedia of Genes and Genomes (KEGG) functional enrichment analysis of DEGs screening out in Fig. 3A (**C**) Lists of statistically significant DEGs between CK and treatment after waterlogging for 5 days and 10 days were used to create a Venn diagram. **D** KEGG functional enrichment analysis of DEGs screening out in Fig. 3C

Next, the gene distribution characteristics between multiple comparison groups were further analyzed using Venn diagrams. First, genes that may not be related to flooding treatment were excluded, and only DEGs found in sensitive and tolerant types after the waterlogging treatment were focused upon. In total, 1,457 and 1,891 specific DEGs were thus discovered at 5 and 10 dat, respectively. In addition, 231 specific DEGs were found in two periods (Fig. 3A). These 3,579 DEGs were further employed for functional annotation analysis. Based on the Kyoto Encyclopedia of Genes and Genomes (KEGG) enrichment analysis, considerable enrichment was observed in several key plant pathways, including brassinosteroid biosynthesis, plant hormone signal transduction, mitogen-activated protein kinase (MAPK) signaling pathways, and isoquinoline alkaloid biosynthesis, among others (Fig. 3B, Table S6). Next, the multiple groups used for comparison between control and treated samples were analyzed and the 4 comparison groups were found to share 673 DEGs among themselves (Fig. 3C). Furthermore, 939 DEGs appeared only between the 2 comparison groups of the waterlogging-tolerant pepper, and 377 DEGs were only identified between the 2 comparison groups of the waterlogging-sensitive pepper (Fig. 3C). KEGG analysis of these 1,989 DEGs revealed considerable enrichment in pathways associated with phenylpropanoid biosynthesis, secondary metabolite biosynthesis, taurine and hypotaurine metabolism, MAPK signaling pathways, and plant hormone signal transduction (Fig. 3D, Table S7).

### Effect of waterlogging on plant physiology

**Fig. 4:**
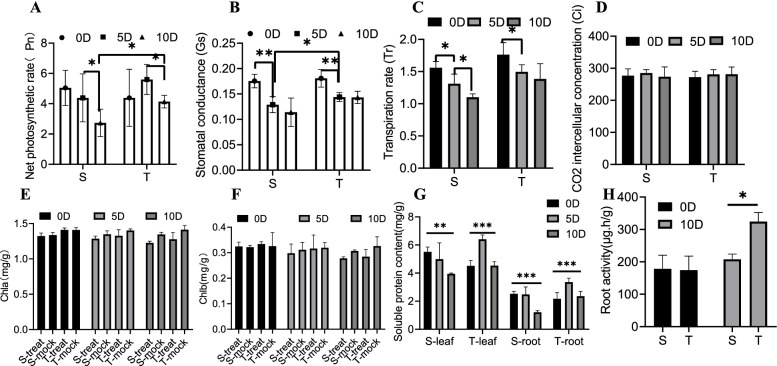
Statistics of physiological indicators after waterlogging. **A** Net photosynthetic rate. **B** Stomatal conductance. **C** Transpiration rate. **D** CO_2_ intercellular concentration. **E** Chlorophyll A content. **F** Chlorophyll B content. **G** Soluble protein content. The effects are significant at *p* < 0.05 using a one-way ANOVA. **H** Root activity. Values are means ± SD (*n* = 3 biological replicates); Asterisks indicate statistically significant differences as determined by Student’s t-test: *, *P* < 0.05; **, *P* < 0.01; ***, *P* < 0.001

Physiological indicators of pepper treatment by waterlogging were also evaluated to determine the molecular pathways fundamentally associated with waterlogging resistance. The findings revealed that the net photosynthetic rate (Pn) of the two varieties of pepper decreased at 10 dat compared with at 5 dat. However, the Pn value of the T variety was noted to be considerably higher than that of the S variety at the same time point (Fig. 4A). Drops in stomatal conductance (Gs) in both varieties of peppers were also observed at 5 dat. In addition, the waterlogging-sensitive variety showed a greater range of variation in Gs and was considerably different than the values recorded for the T variety (Fig. 4B). A significant downwards trend in transpiration rate, which is consistent with the trend in Gs (Fig. 4C), was also noted. However, the intercellular concentration of CO_2_, chlorophyll A content, and chlorophyll B content did not change between the two pepper varieties or among treatment periods (Fig. 4D–F). Additional measurements of soluble protein concentration revealed considerable changes after waterlogging in both treatments, albeit in opposite directions. The S variety exhibited a gradual decline in protein content after waterlogging, whereas the T variety initially exhibited a considerable increase, followed by a decline in the protein content back to the original pre-treatment level (Fig. 4G). Finally, the findings of this study revealed that the waterlogging-tolerant pepper variety had a considerably higher root activity than the waterlogging-sensitive pepper variety at 10 dat (Fig. 4H).

### Enzymatic activity response of pepper to waterlogging stress

**Fig. 5:**
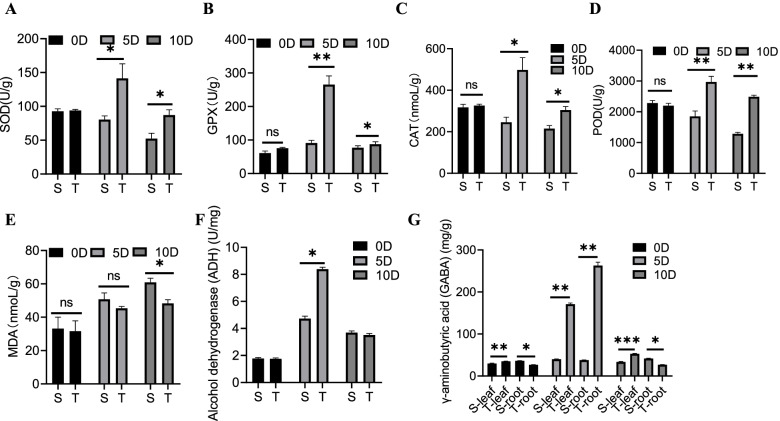
Enzyme activities of pepper under waterlogging. **A** SOD. **B** GPX. **C** CAT. **D** POD. **E** MDA. **F** ADH. **G** GABA. Values are means ± SD (*n* = 3 biological replicates); Asterisks indicate statistically significant differences as determined by Student’s t-test: ns, no significance; *, *P* < 0.05; **, *P* < 0.01; ***, *P* < 0.001

Waterlogging stress can deplete soil oxygen, which can be compensated for by the plant roots by increasing antioxidant enzyme activity. The results obtained revealed that the activities of superoxide dismutase (SOD), glutathione peroxidase (GPX), catalase (CAT), and peroxidase (POD) in S plants were considerably lower than those in T plants at 5 and 10 dat (Fig. 5A–D). Furthermore, there was a considerable decline in the malondialdehyde (MDA) concentration in T plants at 10 dat (Fig. 5E); however, ADH concentration was considerably higher in T than in S plants only at 5 dat (Fig. 5F). These results also revealed that the γ-aminobutyric acid (GABA) concentration in S stems and leaves was lower than that in the same tissues of T plants, whereas the GABA concentration in S roots was higher than that in T roots both before the waterlogging treatment and at 10 dat. The GABA concentration in S plants was considerably lower than that in T plants in both tissues at 5 dat (Fig. 5G).

### Gene expression patterns and DEG clusters

**Fig. 6:**
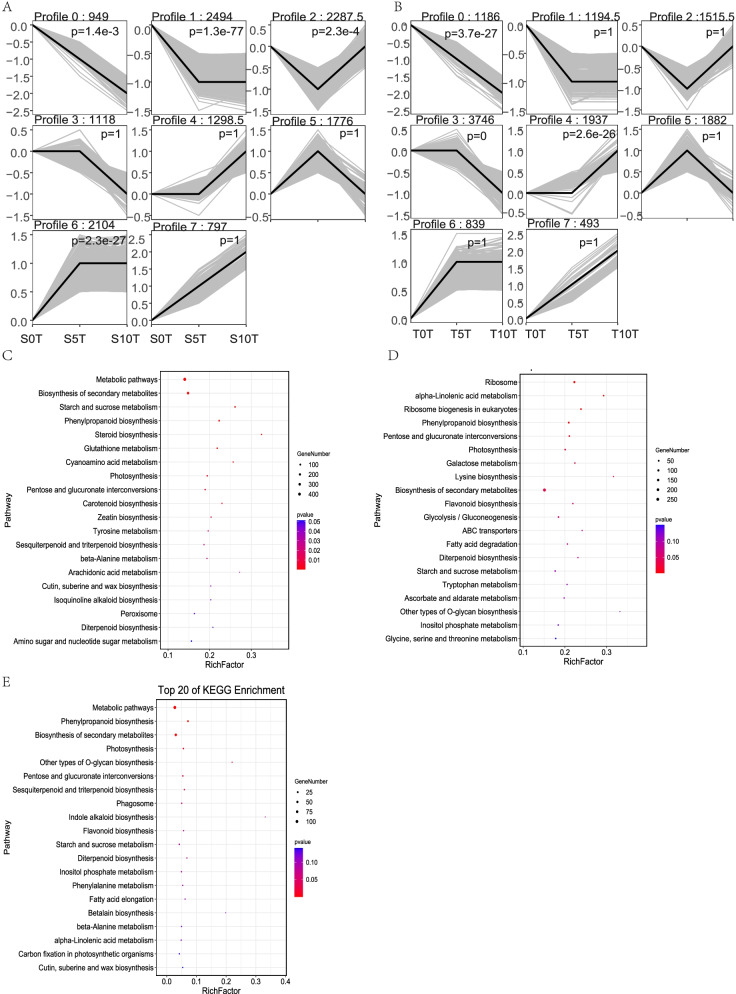
The trend of gene expression after waterlogging by STEM software and KEGG enrichment. **A** The trend of gene expression of S, **B** The trend of gene expression of T, **C** KEGG enrichment of clusters 1 and 6 in S, **D** KEGG enrichment of clusters 3 and 4 in T. **E** KEGG enrichment of cluster 2 and 5 in T. The number on the top of a profile box is the profile ID number and the number of genes expected, and the enrichment *p*-value is in the top right-hand corner of a profile box (significance *p* < 0.05). Y-axis means the expression change compared to the first time point

Next, the clustering of DEGs of T and S plants at different time stages was investigated. Eight distinct profiles were identified based on the gene expression patterns analyzed using the Short Time-series Expression Miner (STEM) software. These profiles showed considerable differences in gene expression over time in response to waterlogging stress between T and S plants (Fig. 6A, B). Initially, profile 0 DEGs were found to be considerably enriched in both S and T plants; however, profile one and profile six were both considerably enriched in S plants (i.e., they were either considerably up- or down-regulated at 5 and 10 dat compared with the pre-treatment). Furthermore, profiles 3 and 4 were noted to be considerably enriched in T plants after waterlogging (exhibiting a slight change at 5 dat but considerable up- or down-regulation at 10 dat; Fig. 6A, B). This suggests that waterlogging has a greater effect on waterlogging-sensitive pepper varieties in the initial period after the onset of the waterlogging stress.

Further analysis of clusters 1 and 6 in S plants showed that these DEGs were considerably enriched in the following KEGG pathways: starch and sucrose metabolism, phenylpropanoid biosynthesis, steroid biosynthesis, glutathione metabolism, cyanoamino acid metabolism, and photosynthesis (Fig. 6C). The considerably enriched KEGG pathways connected with clusters 3 and 4 in T plants included the following: ribosome, alpha-linolenic acid metabolism, ribosome biogenesis in eukaryotes, and phenylpropanoid biosynthesis, pentose and glucuronate interconversions, photosynthesis, galactose metabolism and lysine biosynthesis (Fig. 6D). Next, genes of clusters 1 and 6 in S plants and clusters 3 and 4 in T plants were subjected to Gene Ontology (GO) enrichment analysis. The results showed that the main enriched GO terms for the two sets of DEGs were highly similar (Fig. S1 A, B). Genes that were present in both sets were subjected to further KEGG analysis, and these genes were found to be enriched in the following pathways: phenylpropanoid biosynthesis, biosynthesis of secondary metabolites, photosynthesis, other types of o-glycan biosynthesis, pentose and glucuronate interconversions and sesquiterpenoid and triterpenoid biosynthesis (Table S8).

Furthermore, as stated above, the changes in physiological chemistry emerged at 5 dat, and the activity of antioxidant enzymes was higher in T plants than in S plants. Considering these findings, the DEGs in clusters 2 and 5 showed a consistent trend in T plants after the waterlogging treatment, with transcription initially increasing and then decreasing (Fig. 6B). The functional analysis showed that DEGs in these clusters were primarily connected to photosynthesis, selenocompound metabolism, enzymes with Enzyme Commission numbers, zeatin biosynthesis, transcription factors, plant–pathogen interaction, phenylpropanoid biosynthesis, cytoskeleton proteins and the phosphatidylinositol signaling system (Fig. 6E, Table S9).

### Phenylpropanoid biosynthesis pathways

**Fig. 7:**
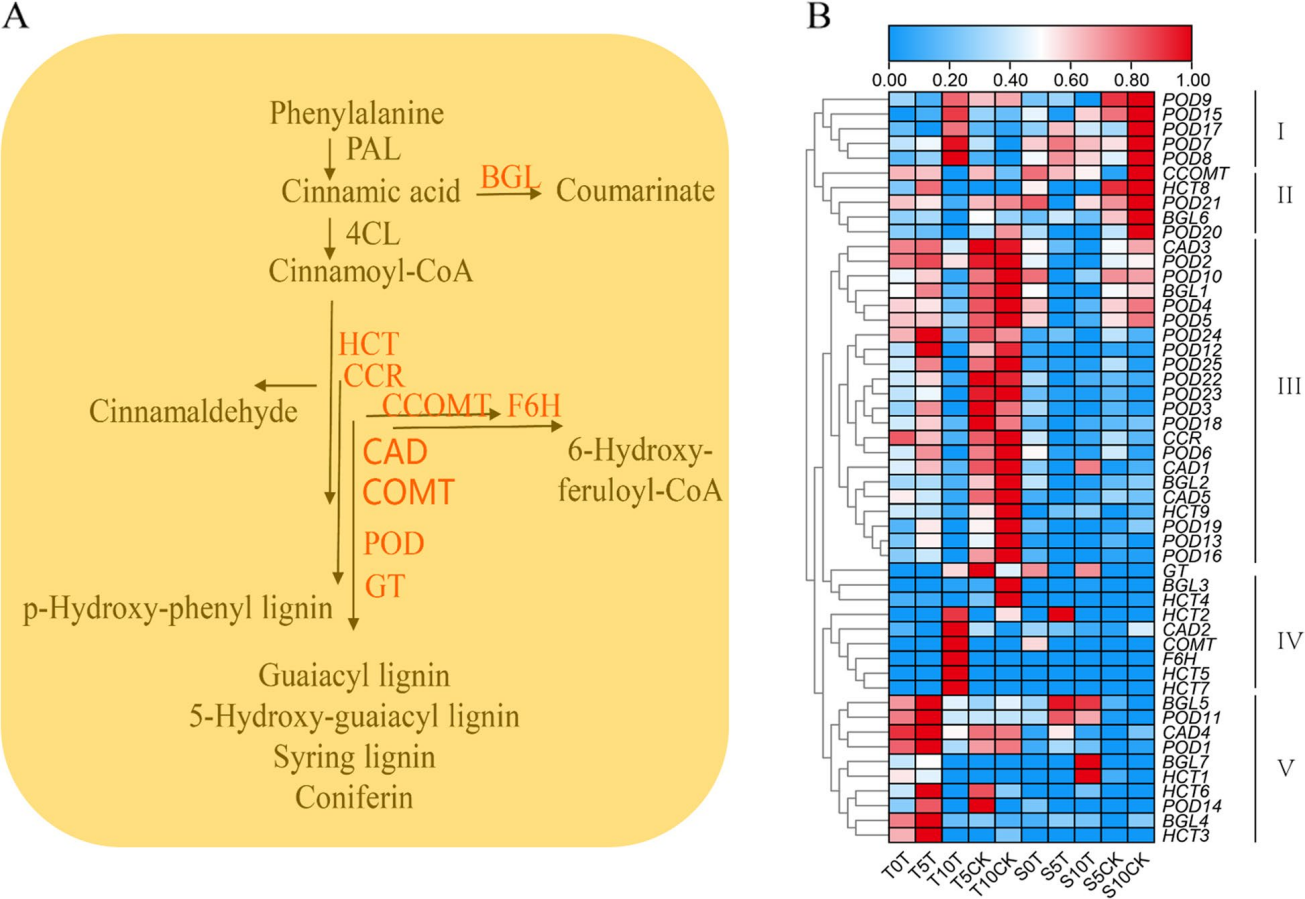
The phenylpropanoid biosynthesis pathway. **A** Phenylpropanoid biosynthesis pathway. POD, peroxidase; BGL, beta glucosidase; CCR, cinnamoyl-CoA reductase; HCT, shikimate O-hydroxycinnamoyl transferase; COMT, caffeic acid 3-O-methyltransferase. CCOMT, caffeoyl-CoA O-methyltransferase; CAD, cinnamyl-alcohol dehydrogenase; F6H, feruloyl-CoA 6-hydroxylase;GT, coniferyl-alcohol glucosyltransferase. Red-letter mean differently expressed genes. **B** The expression profile of genes in the phenylpropanoid biosynthesis pathway. The expression levels are represented by the color bar (log2(FPKM)-transformed and row scale by zero to one method), ranging from low (blue) to high (red) expression

One of the most significant secondary metabolic pathways in plants is phenylpropane metabolism. As stated earlier, a differential regulation of multiple genes in this pathway was noted after pepper flooding stress, particularly in genes associated with the synthesis of various lignins. Significant differences between multiple groups in terms of expression within the flooding treatment were also discovered (Fig. 7A). The expression profiles of genes enriched in phenylpropane metabolism were analyzed, which showed that these genes were split into five clusters. Clusters 1 and 4 were mainly upregulated at 10 dat in the waterlogging-tolerant pepper plants. On the other hand, genes in clusters 2 and 3 were first upregulated and later downregulated in the waterlogging-tolerant pepper plants; however, the expression levels of these genes in the control group gradually increased. The gene expression pattern of cluster 5 was the most consistent with our prior expectations. After the onset of the flooding stress treatment, cluster 5 genes in the T variety were upregulated at 5 dat and downregulated at 10 dat. In contrast, in the S variety, no considerable changes were observed except in the expression levels of beta glucosidase (BGL)5, BGL7, POD11, and shikimate O-hydroxycinnamoyl transferase 1. Generally, it was noted that an increasing expression of multiple POD genes in tolerant peppers at 5 dat following the onset of stress and decreases at 10 dat (Fig. 7B).

### Stress-related pathway

**Fig. 8:**
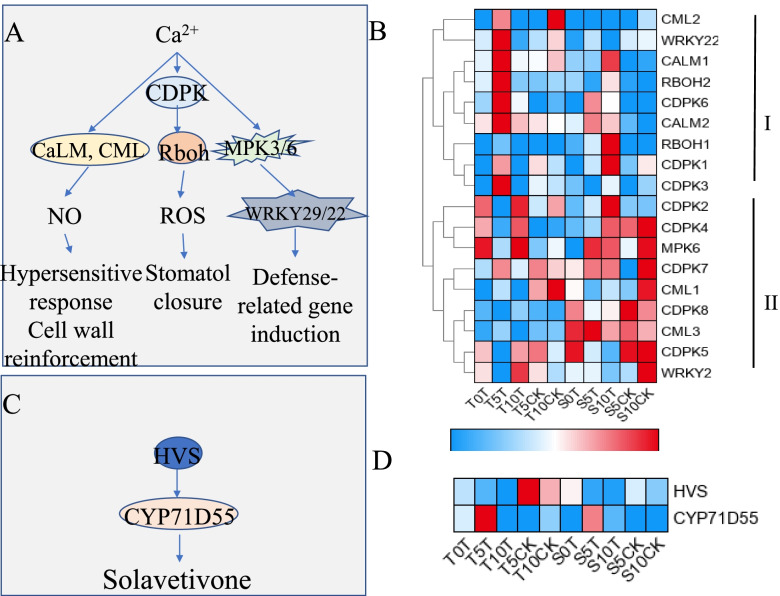
Disease resistance relatedly pathways and gene expression profile. **A** Calcium regulation pathway. **B** The heatmap of DEGs in the calcium regulation pathway. **C** Solavetivone biosynthesis pathway. **D** The heatmap of DEGs in the Solavetivone biosynthesis pathway. The expression levels are represented by the color bar (log2(FPKM)-transformed and row scale by zero to one method), ranging from low (blank) to high (red) expression. CDPK, calcium-dependent protein kinase; CaLM, calmodulin; CML, calcium-binding protein; Rboh, respiratory burst oxidase; MPK6, mitogen-activated protein kinase 6; WRKY22, WRKY transcription factor 22; HVS, vetispiradiene synthase; CYP71D55, premnaspirodiene oxygenase

Pathways associated with disease resistance were considerably enriched, including a pathway associated with calcium ion transport (Fig. 8A). First, *CaLM1*, *CaLM2*, and *CML1-3* were considerably upregulated at 5 dat in the T variety and downregulated afterward, reaching the pre-treatment levels or even lower by the 10^th^ day. This pathway is associated with the nitric oxide concentration present. Second, *CDPK1*, *3*, *6*, and *7* and the downstream genes *RBOH1 and RBOH2* were upregulated at 5 dat and downregulated at 10 dat in the waterlogging-tolerant pepper plants; however, some genes, such as *CDPK2*, *4*, *5*, and *8*, revealed the opposite trend (downregulation at 5 dat and upregulation at 10 dat). These trends may cause changes in ROS levels in the waterlogged plants. Third, the expression levels of MAPK 6 and its downstream gene WRKY transcription factor 22 were both considerably downregulated at 5 dat and upregulated at 10 dat; however, the expression pattern of WRKY22, a gene further downstream, was the opposite. This pathway may trigger the expression of defense-related genes (Fig. 8 A, B).

The changes in calcium ions in the two pepper varieties were also studied. The ion concentrations increased at 5 dat and then decreased at 10 dat, although this change was only noteworthy in the waterlogging-tolerant pepper plants (Fig. S2 A). Furthermore, concentration changes in Mn and Fe ions in the two varieties were examined, and the concentration of Mn ions in the waterlogging-tolerant pepper plants was considerably altered at three time points after the induction of flooding, whereas the concentration of Fe ions changed considerably in the waterlogging-sensitive pepper plants (Fig. S2 B, C).

Finally, considerable variations were noted in the expression levels of genes encoding vetispiradiene synthase *(HVS)* and premnaspirodiene oxygenase *(CYP71D55)* in the waterlogging-tolerant pepper plants. These genes are involved in the solavetivone synthesis pathway. *HVS* was highly expressed in the control group of the T pepper variety but was considerably reduced after the waterlogging treatment. In addition, the expression of its downstream gene *CYP71D55* was considerably upregulated at 5 dat. These findings imply that pepper plants can improve their ability to resist abiotic stress by regulating the synthesis of solavetivone (Fig. 8C, D).

### Transcription factors

Generally, transcription factors have crucial regulatory roles. In the waterlogging-tolerant pepper plants, 51 transcription factors were significantly up- or down-regulated at 5 dat, after which they returned to pre-treatment levels. As indicated in Table S10, these transcription factors include 8 ethylene-responsive transcription factors, 9 homeobox-leucine zipper proteins, 3 heat stress transcription factors, 21 myloblastosis (MYB) transcription factors, 2 WRKY transcription factors, and pathogenesis-related genes such as transcriptional activator PTI5-like (Table S10, Fig. S3).

### RNA-seq expression validation by quantitative real-time polymerase chain reaction (qRT-PCR)

**Fig. 9:**
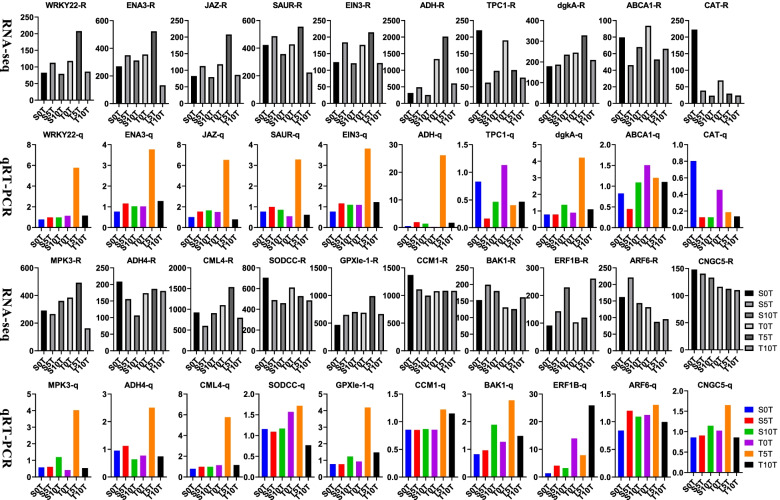
Expression pattern of the selected 20 genes in two treatment peppers. The white and black figures showed the gene expression data of RNA-seq, and the corresponding color figure below showed the gene expression level detect by qRT-PCR

qRT-PCR was used to confirm the reliability of our RNA-seq data. Results of this analysis revealed that of the 20 selected DEGs, the expression patterns of approximately 80% (i.e., all except *superoxide dismutase* (*SODCC)*, *BRASSINOSTEROID INSENSITIVE 1-associated receptor kinase 1(BAK1)*, *AUX/IAA gene* (*ARF6)*, and *cyclic nucleotide-gated ion channel 5* (*CNGC5)*) were consistent with our RNA-seq dataset (Fig. 9). Among these genes, *WRKY22*, *MPK3*, and *CML4* are crucial genes in the MAPK signaling pathway, and their expression levels were upregulated in waterlogging-resistant varieties at 5 dat. Furthermore, the expression levels of *ADH*, *GPXle-1*, and *CAT* genes were highly similar to those of ADH, GPX, and CAT.

## Discussion

Between 1980 and 2021, China’s pepper production doubled. (https://www.fao.org/faostat/zh/#data/QCL). Although peppers are rich in nutrients and well-liked by consumers, they are susceptible to water stress, which can result in plant death and reduced yields after rainstorms. Growing water-tolerant varieties is thus of great value for pepper breeding [23]. Zhang et al*.* obtained a waterlogging-resistant mutant variety “RW15” by EMS and produced a transcriptomic dataset to compare DEGs at 10 dat to identify genes that may be involved in waterlogging stress response. The researchers also attempted to elucidate the mechanisms involved in waterlogging tolerance by employing mutants [24]. In this study, the weight and growth rates were considerably affected at 10 dat, particularly in flood-sensitive pepper plants, although the expression of the affected genes changed considerably earlier than this time point. In this study, to identify the candidate genes of early response to water stress, the effects of a waterlogging treatment on the waterlogging-sensitive “Chuan Teng No*.*6*”* variety and the tolerant “Chuan Teng No.10” variety were compared. To this end, the gene expression at multiple time points after the onset of water stress was measured. The findings of this analysis are shown in Figs. 2 and 3. To further anchor the candidate genes involved in waterlogging, functional enrichment analysis was conducted using these DEGs. The enrichment results were screened by focusing on the changes in biochemical indicators as much as possible.

Following waterlogging, the roots of peppers quickly become anoxic [3]. Anaerobic respiration is utilized to generate oxygen in hypoxic environments where it is difficult or impossible to achieve aerobic respiration. However, this process also produces ethanol as a byproduct [20, 25]. Ethanol inhibits plant growth, although the results of this study showed that the ADH concentration in the waterlogging-tolerant pepper plants was considerably higher than that in their waterlogging-sensitive counterparts at 5 dat, which may have a positive effect on the decomposition of excess ethanol in the former. These results are consistent with the research on tomatoes after waterlogging [20]. Furthermore, the MDA concentration in the waterlogging-tolerant pepper plants was considerably lower than that in the waterlogging-sensitive pepper plants at 10 dat. MDA is an oxidation product generated by the continuous buildup of ROS [26–28]. The ROS-regulated gene Rboh1 and its upstream genes CDPK1, 3, and 6 were upregulated at 5 dat in the waterlogging-tolerant pepper plants. CDPK is a calcium-dependent kinase, and a substantial increase in calcium ion concentration at 5 dat in the waterlogging-tolerant pepper plants was also noted. This evidence implies that this pathway plays an active role in oxygen synthesis. These findings also suggest that in the waterlogging-tolerant pepper plants, oxidative damage may be reduced by decomposing ethanol and calcium ions can regulate the ROS concentration to resist waterlogging.

GABA is a signaling molecule that can enhance the growth of plants subjected to waterlogging. It plays a similar role in maize seedlings and soybean by downregulating ROS and activating antioxidant defenses [13, 29]. In this study, the GABA concentration of the waterlogging-tolerant pepper plants was considerably higher than that of their waterlogging-sensitive counterparts. Because the concentration of GABA was considerably increased in T, it may have led to an increase in the activity of SOD, POD, and CAT and a decrease in the concentration of MDA, and these results are relatively similar to those observed in maize [13].

Regarding the physiological indicator dataset in this study, the Pn value of the two pepper varieties exhibited obvious changes at 5 and 10 dat, whereas the Gs value changed in 0–5 days. Although the change in chlorophyll content was also obvious, it was not directly affected by waterlogging. This is because the flooding treatment first affects the roots, resulting in changes to the secondary metabolism and water transport, which in turn affects photosynthesis.

The phenylpropanoid biosynthesis pathway is a crucial lignin synthesis and metabolism pathway that produces various metabolites, including flavonoids, lignin, lignans, and cinnamic acid amides. Lignin is produced by the oxidative polymerization of lignin monomers, including H-type, G-type, and S-type monomers. Lignin primarily accumulates in the secondary cell wall of plants, where it provides mechanical support to plant tissues and participates in the formation of vessels, water transport, mineral transport, and resistance to abiotic stress [30, 31]. In the waterlogging-tolerant pepper plants, we identified a peak in peroxidase activity that was considerably higher than that observed in the waterlogging-sensitive pepper plants. This peak likely maintained a higher level of antioxidant enzyme activity in the waterlogging-tolerant pepper plants, enhancing resistance to the hypoxic environment caused by flooding [32–34].

Furthermore, the expression of multiple calcium ion-mediated *CML* or *CaML* genes was substantially upregulated on the 5^th^ day in the waterlogging-tolerant pepper plants. These genes may help in reinforcing the cell wall mechanically [35–37]. In addition, changes in calcium ion concentration may lead to the upregulation of *WRKY22* gene expression in the waterlogging-tolerant pepper plants. WRKY22 is a crucial transcriptional regulator associated with the MAPK signaling cascade and in the immune response in many plants [38–41]. This gene triggers the expression of a series of defense-related genes, and this function may be the reason why it is associated with the enrichment of many DEGs in pathways related to environmental information processing (see Fig. 3).

HVS is a homolog of sesquiterpene cyclase and may be associated with the biosynthesis of solavetivone. Solavetivone is a unique metabolite of Solanaceae and is frequently associated with defense responses because it activates defense-related genes [42–44]. As shown before, CYP71D55 (premnaspirodiene oxygenase) is a typical P450 gene that may modulate the expression of solavetivone further upstream. The expression of CYP71D55 was considerably upregulated at 5 dat and was then downregulated at 10 dat. These findings revealed that there may be some stress tolerance mechanisms associated with pepper that are unique to the *Solanaceae*.

Transcription factors modulate multiple pathways and biochemical processes. Here, ethylene-responsive transcription factors and a gene homologous to TOE3, which have been demonstrated to possess functions associated with freezing tolerance, were identified [43, 45]. Furthermore, RAP2 homologs can delay waterlogging by increasing stomatal closure in *Arabidopsis thaliana* [46]. In *Arabidopsis*, two protodermal factor 2 (homeobox-leucine zipper protein) homologs have been found to block potassium channels associated with defense responses [47, 48]. Twenty-one MYB transcription factors were identified; however, only a few members of this family are involved in waterlogging tolerance [49, 50]. Nevertheless, more members of this family may be associated with the waterlogging stress response. Moreover, WRKY transcription factors and the pathogenesis-related transcriptional activator gene PTI5-like have been found to be associated with defense responses in multiple plants [39–41, 51–53]. The fact that these genes were identified through screening implies that these transcription factors were associated with the molecular modulation of pepper waterlogging tolerance at an earlier stage than that observed in previous studies.

In this study, 20 genes were selected for validating the transcriptome data, and the findings were in high agreement with the data obtained from RNA-seq. Among these genes, several genes containing MAPK pathways, such as *WRKY22*, *MPK3*, and *CML4*, demonstrated an upregulated expression in the pepper plants tolerant to waterlogging, further demonstrating that the MAPK pathway may have the ability to improve waterlogging tolerance in pepper. Furthermore, the expression patterns of several biochemical index-related genes, such as *ADH*, *GPXle-1,* and *CAT*, showed changes that were largely consistent with the data generated by biochemical assays.

## Conclusions

In this study, the genetic and physiological changes at different time points after flooding were investigated. A series of physiological indicators and DEGs demonstrated considerable changes by the 5^th^ day after flooding. Waterlogging-tolerant pepper plants may achieve tolerance to flooding by increasing the activity of oxidoreductase. This enhances the tolerance of peppers to unfavorable conditions such as ROS accumulation during flooding. Simultaneously, waterlogging-tolerant pepper plants may also trigger lignin production and the expression of specific tolerance genes. This also shows that the induced expression of anti-waterlogging genes in pepper may occur extremely early, and it is essential to conduct more detailed and in-depth research to identify which genes are activated and when. Furthermore, these two pepper varieties can be further used to cross and construct mapping genetic populations to provide a basis for further mapping of the waterlogging-tolerant genes. In summary, the findings of this study lay a foundation for further analysis of the molecular mechanisms associated with waterlogging tolerance in pepper and provide targets for the molecular breeding of waterlogging resistance in this crop.

## Materials and methods

### Plant material and waterlogging treatments

In this study, two hot pepper varieties (the two varieties were bred by the Horticulture institute of Sichuan Academy of Agricultural Sciences, and their certificate was awarded by the Agricultural Ministry of China), including the S and T varieties were used. First, seeds of the two varieties were surface sterilized by immersion in 70% ethyl alcohol for 1 min. Thereafter, they were rinsed with deionized water. After sterilization, seeds were soaked in deionized water at 28 °C in an incubator for 24 h in the dark, then germinated on a 50-plug tray. Germination took place on a sterilized soil substrate at a controlled temperature (25 °C − 30 °C) under a 16/8 h light/dark cycle in a greenhouse.

At the six-leaf-one-heart phase, healthy seedlings from each variety having a similar size were transplanted into plastic pots (diameter × height = 17.3 cm × 10.3 cm). After 150 days of growth, three mature-stage plants of each variety from three different plastic pots to be used as biological replicates were identified. Potted plants were subjected to waterlogging stress using a dual-pot technique. Plastic pots were placed into a 51 × 35 × 23 cm container. Each larger container contained 18 plastic pots (6 WSP, 6 WTP). A water level of 2 cm above the top of the plastic pot was considered to be a “waterlogging” treatment. Water was supplemented every other day to maintain the water level, and the waterlogging treatment was repeated three times. Based on variety and treatment, the naming of the samples is shown in Table 1. Shoots and roots were harvested from replicates of both varieties that were similar in size. Samples were first washed with deionized water, then frozen in liquid nitrogen (N_2_), and stored at − 80 °C for RNA extraction and physiological tests.

**Table 1:** The samples list

Name	0 day	5 day		10 day	
	Control	Control	Treatment	Control	Treatment
*Chuan teng No.6*	S0CK	S5CK	S5T	S10CK	S10T
*Chuan teng No.10*	T0CK	T5CK	T5T	T10CK	T10T

### RNA extraction, library construction, and sequencing

Total RNA was extracted from the root tissue using the Trizol reagent kit (Invitrogen, Carlsbad, CA, USA) according to the manufacturer’s protocol. rRNA was removed using a Ribo-Zero™ Magnetic Kit (Epicentre, Madison, WI, USA). Second-strand cDNA was synthesized using DNA polymerase I, RNase H, dNTP, and buffer. cDNA fragments were purified using a QiaQuick PCR extraction kit (Qiagen, Venlo, The Netherlands) and then ligated to Illumina sequencing adapters. Ligation products were subjected to size selection by agarose gel electrophoresis. Products were then amplified using PCR and sequenced using an Illumina HiSeq2500 platform. Three biological replicates were performed for each treatment.

### RNA-seq data analysis

Before assembly, adapter sequences were removed from all raw reads. Low-quality reads (i.e., those with over 50% bases with Q-values ≤ 20 and/or over 10% unknown (N) bases) were removed from each data set using fastp to improve reliability [54]. Next, the short reads alignment tool implemented in Bowtie2 [55] (version 2.2.8) was used to map reads to an rRNA database. All rRNA-mapped reads were then removed. The remaining clean reads were used for transcriptome assembly and gene abundance calculations.

The *Capsicum annuum* L. reference genome (version 2.0) and associated annotation files were downloaded from China National GeneBank (accession no. CNPhis0000547; https://ftp.cngb.org/pub/CNSA/data2/CNPhis0000547/pepper/) [56]. An index of the reference genome was built, and paired-end clean reads were then mapped to this genome using HISAT2. 2.4 with “-RNA-strandedness RF” and other parameters set to the default [57]. The reconstruction of transcripts was conducted using Stringtie (version 1.3.1) and HISAT2 [57, 58].

### Gene expression analysis

For each transcription region, Stringtie was employed to calculate an FPKM value to quantify gene expression abundance [58]. DEGs between different samples were then identified using DESeq2 [59]. The significance threshold of the *p*-value was adjusted using the false discovery rate (FDR) to correct for multiple testing. Transcripts with a minimal two-fold difference in expression (|log2 Ratio|≥ 1) at FDR ≤ 0.001 were regarded as DEGs.

Furthermore, to assess gene expression patterns over time within each genotype, the time series of the expression patterns of the two waterlog treatments were examined using STEM version 1.3.8.43 [60]. DEGs belonging to the same cluster were proposed to have similar expression patterns. For each variety, the clustered profiles of DEGs at *p* < 0.05 were considered significantly different from the reference set.

### Functional annotation and GO and KEGG classification

DEGs were then subjected to GO functional analysis and KEGG pathway analysis [61, 62]. For each treatment stage, the *p*-value was corrected using the FDR, with FDR ≤ 0.05 as a threshold. For each KEGG pathway, the numbers of up- and down-regulated genes from each variety were compared with the reference set using a Fisher’s exact test to identify pathways enriched in up- and down-regulated genes.

### Antioxidant enzyme extraction and analysis

Fresh leaf samples (0.1 g) were finely pulverized in 2 mL extraction buffer (50 mM phosphate buffer (pH = 7.8)) containing 1 mM EDTA and 2% (w/v) PVP. Samples were then centrifuged at 10,000 × g for 20 min at 4℃. The crude extract was then transferred to another tube and used for enzyme determination. SOD activity was determined following the method of Giannopolitis and Ries [63], and the POD and CAT activities were analyzed using the processes described by Cakmak and Marschner [34] and Bergmeyer [64], respectively. An MDA POD and CAT Assay Kit was obtained from the Nanjing Institute of Biological Engineering (Nanjing, China). A glutathione peroxidase assay kit was obtained from Geruisi Biotechnology Limited (China), and the kit was used as per the manufacturer’s specifications. The activity of all antioxidant enzymes was determined on a fresh weight basis.

### Photosynthetic activity

Next, plant leaves were collected to determine the concentrations of photosynthetic pigments. Samples (0.5 g of fresh weight per treatment) were extracted in 80% (v/v) methanol and ground thoroughly. The concentration of the supernatant of this mixture was then determined spectrophotometrically (i.e., chlorophyll A (Chl a) at 663 nm, chlorophyll B (Chl b) at 645 nm), as described by Lichtenthaler [65]. The absorbance of chlorophyll in the extract was measured using a Multiscan Go microplate reader (Thermo Fisher Scientific, USA).

Leaf photosynthetic rate, stomatal conductance, transpiration rate, and intercellular CO_2_ concentration were measured using a Li-6400 portable photosynthesis system (Li-Cor, Lincoln, USA). All measurements were performed between 9:30 and 11:30 h. Leaves in the upper part of the plant were selected and labeled for daily leaf measurements.

Root activity was analyzed using the triphenyl tetrazolium chloride (TTC) method [66]. Root activity was expressed as TTC reduction intensity. Root activity = amount of TTC reduction (µg) / fresh root weight (g) × time (h).

### ADH activity assays

After measuring photosynthesis, roots from individual plants were harvested and flash-frozen in liquid nitrogen. ADH enzyme assays were performed according to the method of John and Greenway (1976) to determine the level of root oxygen stress [67]. NADH oxidation was determined by measuring decreases in absorbance at 340 nm. Background rates of substrate reduction were determined in the presence of 80 M NADH with a buffer containing 40 mM bicine and 5 mM MgCl_2_ at a pH of 8.0. Reaction rates were determined following the addition of 10 mM acetaldehyde [68, 69].

### Determination of GABA content

Fresh leaves and roots (1.0 g) were ground in 5 mL of 7% acetic acid. The purification of GABA was performed as described previously [70]. GABA was determined via high-performance liquid chromatography (Agilent 1200, USA) using a ZORBAX Eclipse AAA reversed-phase column (4.6 × 150 mm inner diameter, 3.5 µm particle size) as described previously [29]. The elution program was performed as described previously [71].

### Validation of gene expression patterns

Twenty genes, including MAPK signaling pathway-related genes, and the abovementioned biochemical indicator-related genes, were selected for validation by qRT-PCR. First-strand cDNA was synthesized using a TUREscript 1^st^ Stand cDNA Synthesis Kit (Aidlab, China). Gene-specific primers for qPCR were designed based on the corresponding sequence using Primer3 (https://primer3.ut.ee) and are listed in Table S11. β-tubulin was used as an internal control. qRT-PCR was performed using 2 × SYBR® Green Master Mix (DBI, China) and a qTOWER 2.0/2.2 Quantitative Real-Time PCR Thermal Cycler (Analytik Jena, Germany) according to the manufacturer’s instructions. Three technical replicates were performed for each gene. A regression analysis was performed using qRT-PCR and RNA sequencing results; this analysis included all genes from both genotypes at all the three time points of the waterlogging treatment. Relative gene expression (calculated using the 2^−△△Ct^ method) was visualized graphically.

## Supplementary Information

**Additional file 1: Fig. S1.** GO enrichment analysis. (A) GO enrichment of clusters 1 and 6 in S, (B) GO enrichment of clusters 3 and 4 in T.

**Additional file 2: Fig. S2.** The concentration on ions in pepper roots. (A) Ca^2+^, (B) Mn^2+^, (C) Fe^3+^.

**Additional file 3: Fig. S3.** The expression profile of the screened-out transcription factor. The expression levels are represented by the color bar (log2-transformed and row scale by zero to one method).

**Additional file 4: Table S1.** Data filtering statistics.

**Additional file 5: Table S2.** Statistical of RNA-seq base information.

**Additional file 6: Table S3.** The ribosome mapping statistics.

**Additional file 7: Table S4.** The statistical of mapping the reference genome.

**Additional file 8: Table S5.** The mapping region statistics.

**Additional file 9: Table S6.** The KEGG enrichment of 3,579 specific DEGs in S10T-vs-T10T and S5T-vsT5T.

**Additional file 10: Table S7.** The KEGG enrichment of 1,989 specific DEGs compared the control and treatment samples taken at the same time point.

**Additional file 11: Table S8.** The KEGG enrichment of the intersection genes in the trend of S and T gene expression analysis.

**Additional file 12: Table S9.** The KEGG enrichment of clusters 2 and 5 genes in Fig. 6B.

**Additional file 13: Table S10.** The DEG transcription factor list.

**Additional file 14: Table S11.** The premiers list for qRT-PCR.

## Data Availability

All the RNA-seq raw data used in this study have been deposited at NCBI BioProject ID: PRJNA798101 (http://www.ncbi.nlm.nih.gov/bioproject/798101).

## Abbreviations

ABA: Abscisic acid
ACC: 1-Aminocyclopropane-1-carboxylic acid
ADH: Alcohol dehydrogenase
CAT: Catalase
CML4: Calcium-binding protein 4
DEGs: Differentially expressed genes
GABA: γ-Aminobutyric acid
GPX: Glutathione peroxidase
MPK3: Mitogen-activated protein kinase 3
MAPK: Mitogen-activated protein kinase
MDA: Malondialdehyde
KEGG: Kyoto Encyclopedia of Genes and Genomes
POD: Peroxidase
qRT-PCR: Quantitative reverse transcriptase PCR
SOD: Superoxide dismutase
