# Supplementary figures and images for: Antioxidant activation, cell wall reinforcement, and reactive oxygen species regulation promote resistance to waterlogging stress in hot pepper (Capsicum annuum L.)
Source: BMC Plant Biol. 2022 Sep 1;22:425. doi: 10.1186/s12870-022-03807-2 (PMC9434832; doi:10.1186/s12870-022-03807-2)

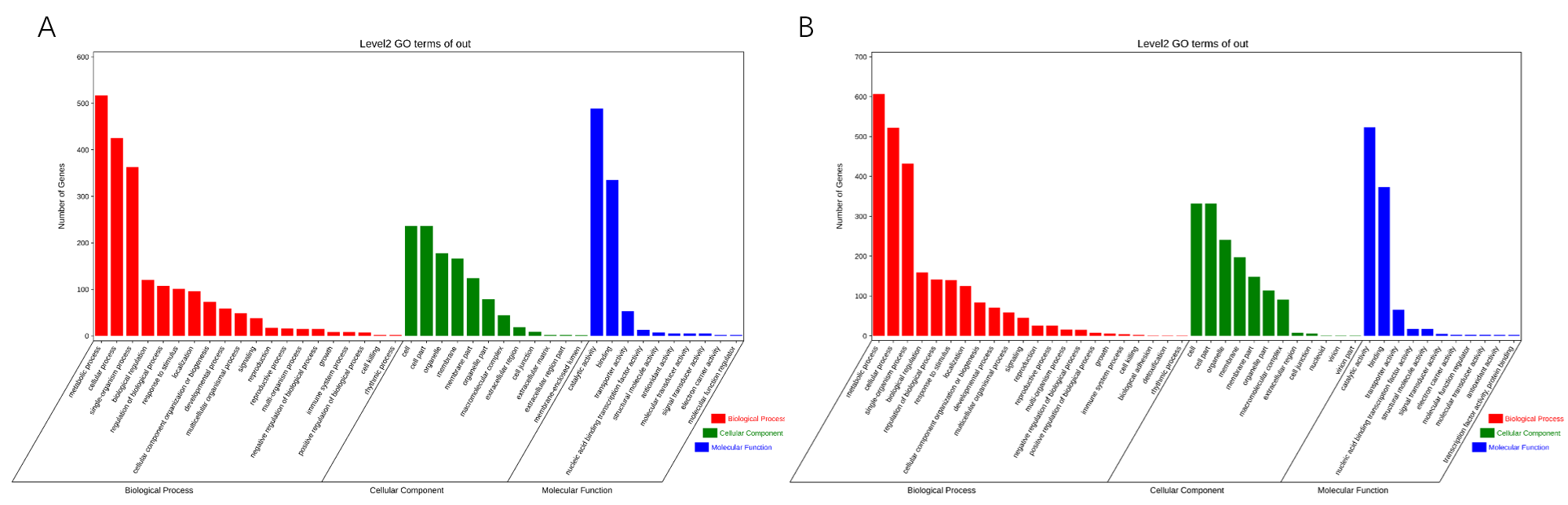

Supplement: Supplementary file 1 — Additional file 1: Fig. S1. GO enrichment analysis. (A) GO enrichment of clusters 1 and 6 in S, (B) GO enrichment of clusters 3 and 4 in T. [file 12870_2022_3807_MOESM1_ESM.tif]

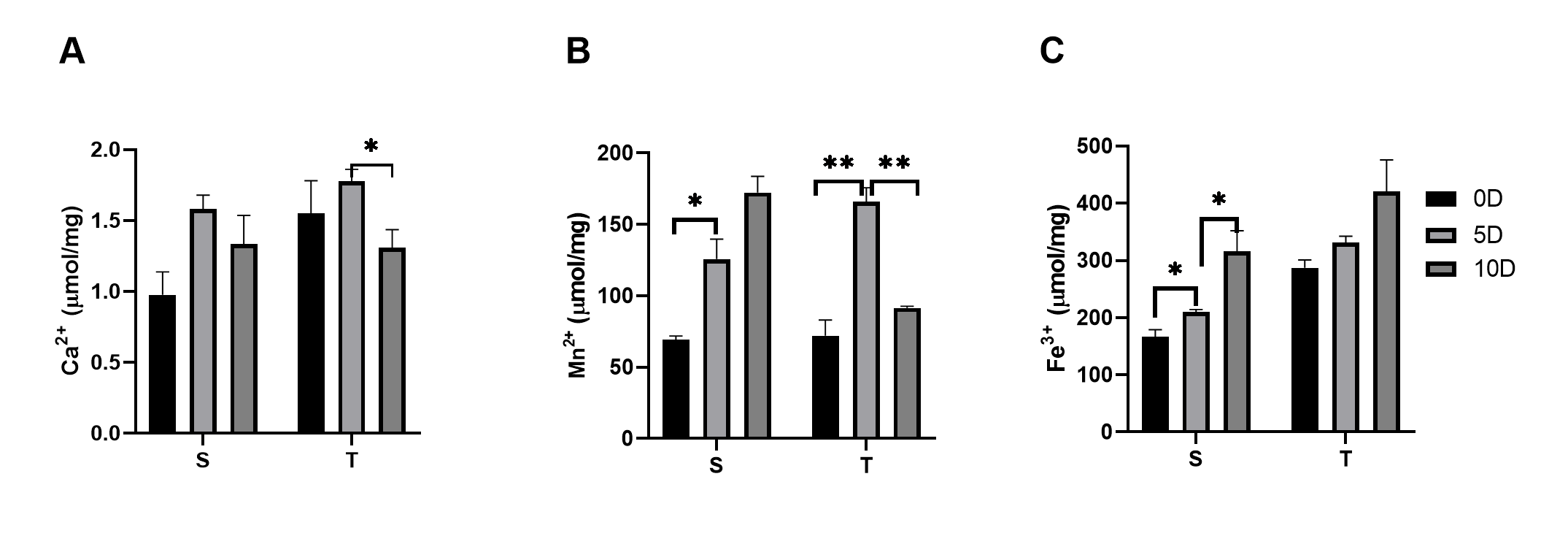

Supplement: Supplementary file 2 — Additional file 2: Fig. S2. The concentration on ions in pepper roots. (A) Ca2+, (B) Mn2+, (C) Fe3+. [file 12870_2022_3807_MOESM2_ESM.tif]

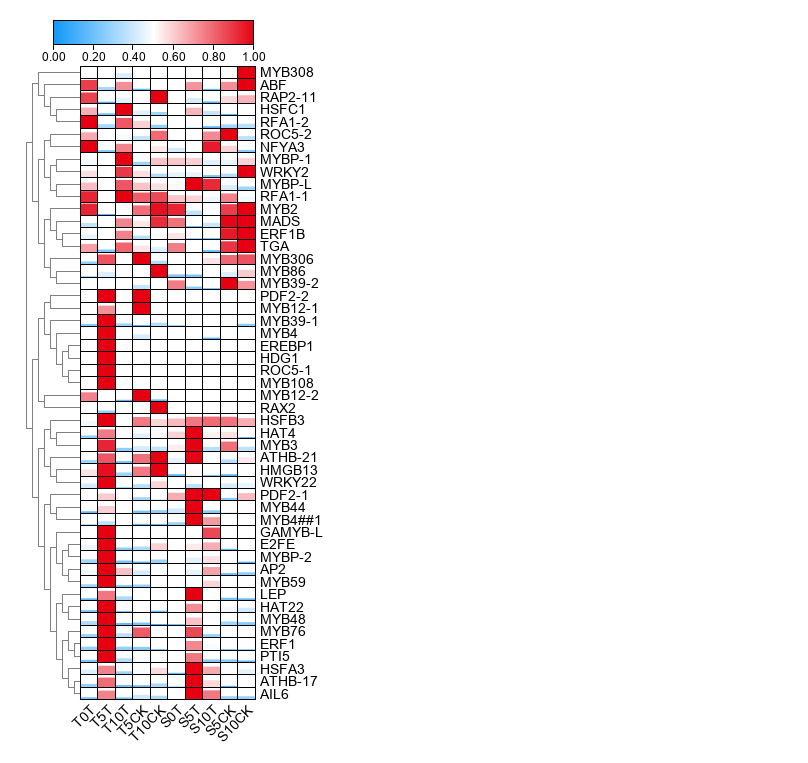

Supplement: Supplementary file 3 — Additional file 3: Fig. S3. The expression profile of the screened-out transcription factor. The expression levels are represented by the color bar (log2-transformed and row scale by zero to one method). [file 12870_2022_3807_MOESM3_ESM.png]
